# Supplementary material for: Are carbon emissions trading and green financial instruments synergistic? -Comprehensive quantitative research based on content analysis
Source: PLoS One. 2024 Mar 7;19(3):e0298601. doi: 10.1371/journal.pone.0298601 (PMC10919601; doi:10.1371/journal.pone.0298601)
Supplement: S1 Data — (ZIP) [file pone.0298601.s001.zip › Data/地级市Z_F工作报告.zip!/║┌┴·╜¡/╝╤─╛╦╣╩╨/└┤╫╘╣1⁄2╓┌║┼í╛┬φ┐╦╩2╛▌═°í┐_2013.docx]

各位代表：

现在，我代表市人民政府向大会报告工作，请予审议，并请各位政协委员和列席会议的同志提出意见。

　　一、2012年工作回顾

　　过去的一年，我们在省委、省政府和市委的正确领导下，团结和带领全市人民，积极应对复杂多变的宏观形势，坚持稳中求进，突出加快发展，以项目建设为牵动，扎实推进九大战略任务，圆满完成市十五届人大一次会议确定的各项目标。全市预计完成地区生产总值660亿元，同比增长13%。完成财政总收入71.6亿元，同比增长16.8%；公共财政预算收入37.9亿元，同比增长27.3%，市本级公共财政预算收入24.6亿元，同比增长27.5%；固定资产投资360亿元，同比增长35%。城镇居民人均可支配收入实现16150元，同比增长16%，农民人均纯收入突破万元大关，同比增长13%以上。

　　——以招商促投资，项目建设成效显著。全市开复工产业项目499项，同比增加171项，完成投资220亿元，同比增长50.1%，占固定资产投资总额的61.1%。其中，亿元以上项目95个，完成投资108亿元，同比增长40%。列入省重点推进的38个产业项目全部开工并超额完成年度投资计划。山东泉林秸秆综合利用等一批投资规模大、产业带动力强的重大项目启动建设，哈佳快速铁路等一批关系我市长远发展的重大基础设施项目前期取得突破性进展。全年预计招商引资到位资金275亿元，同比增长40%，实际利用外资1.74亿美元，同比增长20%。全市重点建设的十大产业园区新征土地656.3公顷，基础设施投入10.6亿元，新开工产业项目61项，完成投资68.5亿元，成为招商引资的主阵地，产业集聚的大平台。

　　——转方式促增效，工业运行质态良好。坚持抓大不放小，规模以上工业增加值预计完成106亿元，同比增长23%。新增中小企业543户，新进规模以上工业企业13户。全市有2户企业晋升为国家级高新技术企业，完成新产品开发21项，获省级奖励11项，其中省科技进步一等奖1项，应用类科技成果转化率达75%以上。高新技术产业产值预计实现207亿元，同比增长27%。

　　——强基础促增产，现代农业快步迈进。新增水稻种植面积130万亩、玉米种植面积108万亩，总面积分别达到632万亩和678万亩。蔬菜种植面积68万亩，商品量85万吨，同比增长18%。农业良种覆盖率达到98%以上，三大粮食作物标准化栽培面积达到1494万亩。新增千万元以上农机合作社30个，农机总动力达到345万千瓦，综合机械化程度达到91.3%。新发展农民专业合作社179个，总数达到859个，辐射带动52%的农户实现规范化经营。新增场县合作共建面积90万亩，总面积扩大到530万亩，技术辐射面积1360万亩。新建续建大型灌区4座，灌区新增灌溉能力35万亩。全市粮食总产预计突破160亿斤，牧业、渔业、林业产值同比分别增长24.4%、10.5%和20.6%。全国首次现代农业建设交流现场会在我市成功召开。

　　——求合作促开放，发展活力日益显现。成功举办第三届中俄农机展和境外俄罗斯犹太自治州农机展，创造“境内展会—长期展示—境外互动”的展会发展新模式。全国惟一的农作物收割机械设备质量监督检验中心顺利通过国家审核，启动运行。组织开展与意大利、韩国、日本、俄罗斯等国在多领域的交流互访，成果显著，与广东省惠州市结成经贸合作伙伴城市。完成佳木斯机场跑道配套工程并投入使用，佳木斯—北京、佳木斯—上海航班更换机型，班次增加。新开通佳木斯—大连—三亚、佳木斯—哈巴罗夫斯克航线，机场每周起降客运班次达51班，全年客运量突破30万人次。成功举办四大节会，实现“一季游”向“四季游”延伸，旅游收入11.5亿元，同比增长16%。城乡市场繁荣活跃，预计全年实现社会消费品零售总额276.5亿元，同比增长15.5%。全市新发展3家融资性担保公司和6户小额贷款公司，首家村镇银行桦川融兴村镇银行正式开业。金融机构预计存贷比达到69.4%，同比提高6.2个百分点。

　　——重统筹促互动，城乡建设步伐加快。城市控制性详细规划实现全覆盖，完成镇村空间布局规划和村屯规划编制方案。城区重点建设主干道路16条，城市框架进一步拉开。翻建改造区街巷道41条，维修道路37条，中心城区主干道路亮灯率达到98%以上，两侧硬覆盖率达到100%。开展违法建筑综合整治，拆除违章建筑面积13万平方米。加强环卫监督和绩效管理，城市环境卫生明显改善。投入1200万元清雪经费和4000万元设备，市区主要街路机械化清雪率达到80%以上。城区取缔非法营运残疾人代步车，人力客运三轮车平稳退出市场，新建公交站亭271个。全市小城镇当年完成投资9.7亿元，其中7个百镇试点完成投资5.6亿元，建成四星、五星级村42个。100%乡镇、行政村和90%自然村屯通上水泥路，农村砖瓦化率达到80%以上。国家环保模范城、园林城创建工作扎实推进，全面完成年度主要污染物减排任务，我市获得全国减排工作先进集体荣誉。市区好于二级以上空气质量天数达到350天。松花江流域污染防治项目进展顺利，市区东、西城区和5个县（市）污水处理厂全部运行并通过验收，松花江佳木斯段好于三类水质上升25%。创建国家和省级生态乡镇7个、生态村37个。植树造林30.2万亩，全市森林覆盖率、城区绿化覆盖率分别达到15.4%和40.4%，连续26年无重特大森林火灾。

　　——惠民生促和谐，人民生活持续改善。全市民生投入95亿元，市本级投入35亿元，年初确定的30件民生实事当年完成23件，完成年度指标7件。全市新增就业人数完成年计划的116%，城镇登记失业率控制在4.16%；小额担保贷款投放1.4亿元，扶持小微企业5158户，新增创业小老板5000人，我市被命名为全国创业先进城市。实现城乡养老保险制度全覆盖，失业、医疗、新农保参保人数超额完成年度计划；城镇职工基本医疗保险、大额医疗救助和意外伤害保险统筹基金年最高支付限额分别达到5万元、20万元和3万元；城市低保标准提高到310元/月，农村低保标准提高到2000元/年，均超过省规定标准。住房公积金当年归集额8.1亿元。免费享受优质高中教育资源覆盖面逐步扩大，学生交通安全、校园安全及周边环境明显改善，城区中小学20座旱厕全部改为水厕；新建、改扩建公办幼儿园36所；成功探索大职教办学模式，得到省委、省政府和教育部的充分肯定。启动与全国名医名院“五联”工程，市民在本市即可享受优质医疗服务，社区卫生服务能力大幅提升，新农合参合率达到99.4%。人口计生奖扶特扶政策全面落实。市区多渠道投入资金34.4亿元，当年竣工保障性安居工程12303套。完成泥草房改造1.7万户、134万平方米，超额完成省计划任务。科技三项经费、城区派出所水电热费用和教育专项资金、文化及产业发展基金、平安佳木斯基金全部纳入市级综合预算。新建区域热水锅炉4台，开展小区建筑节能改造119万平方米；对裕民小区等17个旧小区实施改造和应急维修，全面推行物业服务站新模式，城区弃管楼房实现物业管理全覆盖；新增天然气用户1.5万户，天然气普及率由47%提高到55%。提前实现“十二五”期间农村广播电视“村村通”目标，多项群众文化作品夺得省级和国家级比赛大奖。我市获得全国群众体育工作先进单位、黑龙江省唯一的全民健身城荣誉，快乐舞步健身操成为全国推广的健身项目。依法打击各类犯罪活动，社会治安综合治理继续强化。社会调处体系和信访维稳机制进一步健全，积极化解多件信访积案，应急管理工作不断加强。投入2000余万元在城区配备一台百米消防举高车，全年无重特大安全生产事故。“食品安全城”创建工作取得成效，严肃查处流入我市的毒羊和伪劣猪肉等案件，我市被中国城市竞争力研究会评为“2012中国食品安全十佳城市”。药品稽查和药品不良反应监测工作走在全国前列，居全省第一位。完成清真寺复建工程。全国双拥模范城实现“七连冠”。统计、审计、气象、地震、人防、边防、民族等工作得到加强，妇女儿童、红十字、残联等工作扎实推进。

　　各位代表！过去的一年，我们坚持科学、民主、依法行政，自觉接受人大法律监督、政协民主监督、社会舆论监督，主动听取民主党派、工商联和社会各界意见和建议，年度人大代表建议和政协提案办结率实现100%。加强勤政廉政建设，推进行政监察、政务公开、行风评议，房地产开发建设领域违规问题、“小金库”等专项治理工作取得新进展。向城区下放第二批行政权力，清理许可和非许可项目617项，压缩精减113项，各类审批事项限时办结率达到100%。建立便捷畅通的政民互通渠道，一批影响发展环境和群众利益的热点、难点问题得到有效解决，多件历史遗留问题得到妥善处理。

　　各位代表，回顾过去一年的奋斗历程，我们在积极面对困难中抢抓机遇，迎接挑战，取得了令人欣喜的成绩。这是省委、省政府和市委统揽全局、正确领导的结果，是市人大、市政协加强监督、鼎力支持的结果，是各条战线励精图治、不懈努力的结果，是全市上下团结奋斗、拼搏进取的结果。在此，我代表市人民政府，向全市人民，向各民主党派、工商联、人民团体，向驻佳部队和武警官兵，向中省直驻佳单位及所有关心、支持佳木斯发展的各界人士以及海内外佳木斯人表示崇高的敬意和衷心的感谢！

　　在取得成绩的同时，我们也清醒地看到，我市经济社会发展中还存在着不容忽视的问题。主要表现在：经济总量小，财政实力弱，城乡公共服务和社会保障水平还不高；产业结构不尽合理，整体竞争力不强，经济发展的质量和效益有待进一步提升；城市管理水平还需提高，一些历史遗留问题还有待进一步解决；政府工作效率效能及廉政建设仍需强化，经济社会发展环境有待进一步优化。面对这些问题，我们要高度重视，采取有效措施加以解决。

　　二、2013年主要工作

　　2013年，是我市实施“十二五”规划承前启后的关键一年，也是为全面建成小康社会奠定坚实基础的重要一年。面对日益繁重的改革发展稳定任务，面对日趋激烈的区域竞争格局，我们必须进一步增强机遇意识和忧患意识，凝聚各方面的智慧和力量，牢牢把握发展的主动权。

　　2013年政府工作指导思想是：以党的十八大精神为指导，以科学发展为主题，以加快转变经济发展方式为主线，以提高经济增长质量和效益为中心，贯彻落实省委、省政府“八大经济区”和“十大工程”建设战略部署，继续突出四大优势，扎实推进九大战略任务，全面加快富裕文明和谐幸福佳木斯建设步伐。

　　今年全市经济社会发展主要目标是：地区生产总值增长12%以上，全社会固定资产投资增长30%以上，公共财政预算收入增长18%以上，城镇居民人均可支配收入和农村居民人均纯收入均增长13%以上，城乡建设、民生保障、生态保护等工作进一步加强，社会公共服务和社会管理水平明显提升。

　　今年我们将重点抓好以下七方面工作。

　　（一）强化项目牵动，调整优化产业结构

　　着力提升产业发展层次和整体竞争力，为长远发展奠定基础，积累后劲。以狠抓项目建设推进产业壮大，做优增量。全年组织国内外招商活动8次，集中签约4次，国内招商引资到位资金计划实现360亿元，同比增长30%,实际利用外资计划实现2.1亿美元，同比增长20%。谋划推进产业项目600项，新开工300项，其中，亿元以上50项，完成投资300亿元，同比增长35%以上。突出抓好列入省重点推进的27个产业项目，确保完成投资目标。加快已开工项目建设进度，促其早日达产达效。继续做好哈佳快速铁路、同江中俄跨江铁路大桥等重大基础设施项目前期工作，力争年内开工建设。以创新驱动推进产业提效，做强存量。全市规模以上工业增加值同比增长18%以上。重点加快94户亿元以上骨干企业扩产改造步伐，培育发展生物制药、新能源和新材料三个新兴产业，积极运用高新技术和先进适用技术，促进装备制造、医药化工、绿色食品等支柱产业向产业链两端延伸，实现传统产业自主创新能力、先进技术装备比重、产品品牌经济比重、产业集约发展水平、经济运行质量效益明显提升。深入实施专利优势企业创建工程，鼓励申请发明专利，新增专利优势培育和示范企业30家，申报国家级高新技术企业5户，认定高新技术产品20项，全市高新技术产业产值同比增长25%以上。推动大企业与知名院校共建工程（技术）研究中心和重点实验室5个。整合资源，调动各方面积极性，充分发挥科技孵化平台作用。以园区优化推进产业集聚，提高质量。调整完善各级园区发展规划，加强园区基础设施和公共服务配套建设，实现布局合理、用地节约、功能明确、要素集聚。

　　（二）提升现代农业，力促农民增产增收

　　发挥资源优势，做优做强农业产业。优化调整种植结构。全市新增水稻80万亩，新增玉米30万亩，总面积均达到700万亩以上。新建城郊型绿色蔬菜生产基地3个，蔬菜面积达到72万亩，产量实现120万吨，商品量90万吨以上。发展农村多种经营。牧业、渔业、林业产值分别增长10%、9.5%、15%以上，新发展大果榛子2万亩，木耳培植量达到6亿袋。加强农超对接，促进农林产品直销入市。扩大境外农业规模，对俄种植面积达到120万亩，实现劳务收入7500万元。加强农业科技推广。建设现代农业示范园区（带）100万亩以上，标准化种植达到80%以上，良种覆盖率稳定在98%以上，实现粮食增产5%。新建水稻智能化浸种催芽基地22处，总数达到67处。场县共建面积新增70万亩，总面积突破600万亩。从种子到田间管理及收获实现安全质量全程监管，推动农业生态、健康、安全、品牌发展。促进农民合作经营。全市新发展农民专业合作社100个，重点培养30个星级社、示范社。示范引导农民带地入社，规模经营土地面积稳定在1000万亩以上。强化基础设施建设。加快临江、三村、引汤等6大灌区建设进度，新增灌溉能力30万亩以上。建立健全农业基础信息数据库，完善农业气象信息预警平台，所有村屯实现气象扬声广播全覆盖。

　　（三）深化改革开放，构筑综合竞争优势

　　以市行政服务及公共资源交易中心建设为牵动，着力打造流程最简、效率最高、收费最低、服务最优、全省一流的行政审批服务平台。在法律、法规和保证城市安全及市民权益的前提下，积极探索社会公共服务市场化运作模式。健全公共财政管理体系，深化国库集中收付等管理制度改革，加强专项资金整合，集中财力办大事。扎实稳妥推进国有文艺院团、报社媒体、厂办大集体和医药卫生体制改革工作。进一步与国际友城开展政府和企业层面的交流互访，争取与澳大利亚、法国、塞内加尔等国有关城市缔结国际友好城市。启动富锦—哈巴罗夫斯克科技园区建设，加快比罗比詹中国农机展示交易中心二期建设，完成比罗比詹哈里特物流园区建设。加快推进同江东港客货通道及服务设施项目。支持引导企业“走出去”，境外投资企业达到35户，实现收入7000万美元。全力办好第四届中俄（佳木斯）农机产品展销洽谈会、犹太自治州境外农机展和我市“四大节会”，拉动会展经济扩大规模。加快推动佳天国际农副产品交易中心二期等现代物流项目竣工投产，促进现代物流业集聚壮大。引领带动经济圈内消费群体的消费理念和行为，社会消费品零售总额增长15%以上。整合域内旅游资源，提升旅游景点品位，积极开拓新航线，推动旅游与贸易融合发展，旅游总收入增长18%以上。规范农机、工程机械、汽车、煤炭等市场秩序，提高管理效益。支持金融机构发展，提高保险业保障能力。争取域外金融机构在我市设立分支机构，推动小额贷款公司试点工作县域全覆盖。加快筹建村镇银行、农村商业银行等新型农村金融机构，积极探索发展农村互助合作金融模式。加强对各类融资担保公司、小额贷款公司监管，防范金融风险。全市金融机构新增贷款力争实现100亿元。

　　（四）加强引导服务，壮大扶优民营经济

　　认真落实国家鼓励和引导民间资本健康发展的有关政策，促进民间资本规范有序进入实体经济领域，民营经济增加值增长23%以上。开展全民创业。在各县（市）区积极引导能人创业、知识创业、青年创业、劳务创业、民生创业、二次创业，降低市场准入门槛，全市新增小微企业1500户，新增规模以上企业41户，中小企业计划实现营业总收入850亿元，同比增长22%。强化引进带动。加大引智、引技、引资力度，在“走出去、引进来”互动融合上下功夫，大力发展文化创意、服务外包、信息咨询、信息处理、专业培训、中介服务等业态，推动民营经济全方位竞相发展。强化政府推动。充分发挥中小企业服务平台和行业协会作用，组织论坛启慧交流，推动银企对接合作，实现对中小企业关爱行动常态化、全程服务亲情化，不断优化发展环境。重点保障有发展前景、资金周转困难的小微企业，积极支持76家成长型中小企业与大企业开展配套合作，加速向集群集聚方向发展。在全市大力弘扬奋发进取、自强自立的主流创业意识，摒弃小富即安、求稳怕险的保守观念，营造尊重创业、尊重创造的良好氛围。

　　（五）坚持统筹兼顾，推动城乡协调发展

　　以新型城镇化为导向，以区域中心城市和生态文明建设为重点，着力建设秀美靓丽佳木斯。组织好第四轮城市总体规划修编，完成城乡供排水、供热燃气、道路建设等修建性规划，实现体系规划、建设规划、土地利用总体规划有机衔接。完成红旗街南延，长安路、胜利路、解放路西延道路和四座桥梁建设，启动双合路、胜利路、学府路铁路跨线桥工程，完成城区桥栏维修改造。抓好城区道路维修，城区道路完好率达到98%以上。实施道路照明精细化管理，街巷、公园亮灯率达到98%以上。细化巷道路网格局，道路通达率达到99%以上。坚持城镇化与新农村建设统筹谋划，各县（市）和郊区、东风区至少确定一个整乡（镇）推进城镇化工作试点，新建一个规模在1000户以上的新型农民社区，建成一个中心镇、两个中心村。完善城区两级执法管理体制，探索推行数字化、网格化管理模式，集中开展标准化工地、环境卫生、私搭乱建、建筑残土、“三废”出城、裸地覆盖、占道经营、门前三包等专项整治行动。继续加大交通整顿力度，加强对公交车、出租车的监督管理，严厉打击非法营运行为。加强国家园林城、环保模范城创建工作，突出抓好松花江水污染防治、主要污染物减排，并以农村环境综合整治为重点，全面加强农村农业面源污染治理。启动城区内河整治工程和PM2.5监测工作。创建国家级生态乡镇、生态村各1个，省级生态乡镇4个、生态村20个。着力抓好市区及县乡主要道路两侧、农村水渠周围绿化，植树造林15万亩，城区绿化覆盖率达到41%。

　　（六）增进民生福祉，促进社会和谐进步

　　探索为民办实事的长效机制，切实让全市人民共享改革发展成果。（1）社会保险参保扩面4万人，探索推动社保卡在社会保障业务中广泛应用。（2）加大对下岗职工和贫困村屯创业致富贷款投入，市区小额担保贷款投放1亿元，贷款额度提高到5万元，对符合条件的高校毕业生放宽到10万元。统筹推进就业技能、岗位技能和创业培训9万人。深入实施城市“一帮一”活动，扶持困难及弱势群体生活就业。全市实现城镇新就业6.6万人，登记失业率控制在4.5%以内。（3）转移劳动力35.5万人，实现劳务收入29亿元。（4）探索建立联合监督执法机制，解决农民工遭拖欠工资、打工者遭恶意欠薪等问题。（5）做好扶贫开发重点村的整村推进工作，扶持贫困户人均年收入超过3000元。（6）解决农村3.6万人饮水安全问题，农村自来水入户率达到80%以上。（7）将城乡低收入群体中的重病重残人员、60岁以上高龄老人纳入医疗救助“一站式”服务工作范围。（8）各县（市）区至少启动一个居家养老试点，鼓励支持民间资本兴办养老、托老机构，引导失能、半失能及低保老人入住养老，各级财政给予定额补贴。（9）市区及各县（市）集中统一、公平分配廉租房和公租房，不搞指标分配区域平均，对老劳模、遗属等人群给予倾斜。（10）关注精神病人、自闭症群体、无救助人群的生活冷暖，开通社会救助绿色通道。（11）合理配置资源，优化教育布局，促进教育均衡发展，教育经费投入达到公共预算的15%。优先改造薄弱学校，完成特殊教育中心学校实训综合楼建设。取消进城务工人员子女义务教育阶段就读户籍限制。（12）完成33所新建、改扩建公办幼儿园，鼓励和扶持民办幼儿园发展。加强培养培训，提高幼儿园教师及保育员配备水平，幼儿园标准化建设水平不断提高。（13）完善“先住院、后结算”模式，低保人群全面推行“一卡通”收费方式。（14）深入开展名医名院医疗合作工程，让群众享受到更优更大的实惠。发挥我市中医优势，开办国医馆。（15）加强社区卫生服务中心规范化建设和支持力度，城乡居民电子健康档案规范化率达到70%以上，建立社区卫生服务中心与市直医疗机构转诊制度。（16）实现城镇医疗保险市级统筹，新农合参合率达到99.6%以上。将全市行政村现有1名村医工资纳入县（市）区预算。（17）启动文化名城建设，继续安排500万元文化及产业发展基金，推出更多群众喜闻乐见的优秀作品，发展小剧场演出等文化形式。（18）推进农村广播电视由“村村通”向“户户通”延伸，文化馆、图书馆、博物馆等公共文化设施免费开放到位。在北京民族文化宫举办佳木斯赫哲文化周活动。（19）积极争创全国全民健身城。规划建设集中、大面积冰场，供中小学生和市民开展冰上活动。维修并新建所有公园及休闲场所的体育健身器械。进一步提高全市体育场馆利用率，满足群众健身需求。（20）启动房产产权交易有形市场，加强对房地产开发经营全过程监管，建立房产市场销售、质量、回迁保证监督体系，保护购房者合法权益。（21）将旧小区应急维修资金列入本级预算，利用三年时间全部完成城区旧小区改造任务。（22）提高小区物业服务管理水平，向市民公开物业收费使用情况。建立高层住宅电梯维修检测管理常态化机制，确保居民乘用安全。（23）市区新增供热面积500万平方米，集中供热率由75%提高到78%，实现市区中小学、托老所、养老院、医疗机构供热达标。（24）将城区内危楼改造、外墙保温、雨中送伞、外立面修整四项工作整体推进，提高市民居住质量。（25）维修改造市区45条主次干路及40条巷道，两年内完成全部巷道改造任务。中心城区和县（市）城关镇裸地处达到100%硬覆盖或绿化覆盖。对城区公园提档升级，江边景区规范管理到位。建设改造城区公厕29座。（26）优先发展城市公交，科学合理地调整、延伸公交线路，加密高峰时段运行班次，实现公交车运行线路无盲区。适当增加市区公交车和出租车容量。（27）合理布局停车场点，规范明示车行路线及停车标识，进一步解决城区停车难和交通拥堵问题。（28）加大社区硬件投入，强化目标管理，建立提高社区管理者待遇和办公经费的增长机制。（29）加快应急指挥平台建设，提高突发事件预防预警、应急处置能力。全面启动平安佳木斯天眼工程，实现城区监控全覆盖，两抢一盗发案率明显下降。加强安全生产管理和监督，有效防范和坚决遏制重特大事故发生。继续完善社会矛盾调处机制，及时解决群众的合理诉求。（30）加大联合执法力度，加快“食品安全城”创建步伐，重点抓好餐厨垃圾无害化处理场项目及餐饮场所达标建设，严厉打击各类食品生产经营违规违法行为，保障市民饮食安全。

　　（七）加强自身建设，打造人民满意政府

　　全面建成小康社会的实践，对政府自身建设提出更新更高要求。我们必须以发展的视角和责任来改进政府工作，以求实的作风和措施来提升政府工作，以法治的监督和规范来保证政府工作。注重在加强学习中解放思想，善于用改革创新的思路寻求解决问题的办法，以勤于实践的作风提高加快发展的能力。坚持把群众需求作为引领政府工作的根本导向，把人民满意作为评判政府工作的重要标尺，从群众最不满意的地方改起，从群众最期盼的事情做起，使政府工作更加契合民情民意。全面推行政府系统绩效管理，实现目标、责任、绩效相统一，推动政府工作提速、提质、提效，坚决遏制行政不作为、乱作为现象。规范公务员技能人才库管理，强化技能日常培训和公务员岗位交流力度，最大限度发挥公务员的积极性和创造力。建立政府重大问题专家咨询系统，完善公众参与、专家咨询和政府决策相结合的科学民主决策机制。加大政府信息公开力度，确保政民互动沟通渠道畅通。加快诚信体系建设，维护良好的社会诚信秩序。牢固树立制度面前没有特权、制度约束没有例外的观念，自觉将政府工作置于人大的法律监督、工作监督，政协的民主监督和公众社会舆论监督之下，有效加强行政监察和审计监督，不断提高对重点领域、关键部位、特殊岗位的监管水平。坚决反对铺张浪费，切实降低行政管理运行成本，实现“四项经费”预算零增长。认真落实党风廉政建设责任制，依法查办各类违纪违法案件，保证权力在阳光下运行。

　　各位代表，新的一年开启了新的征程，新的目标孕育着新的收获。让我们在市委的正确领导下，进一步解放思想，开拓创新，求真务实，团结奋斗，以踏踏实实的努力，换取实实在在的成果，让百姓获得真真正正的实惠，在全面建成小康社会的道路上，奋力谱写建设富裕文明和谐幸福佳木斯的新篇章！
